# A baby presenting with failure to thrive in primary care: a case report

**DOI:** 10.1186/1757-1626-2-137

**Published:** 2009-02-11

**Authors:** Karen J Hoare

**Affiliations:** 1 General Practice and Primary Health Care, School of Population Health and School of Nursing, The University of Auckland, Private bag 92019, Auckland, New Zealand

## Abstract

**Background:**

A nine month old baby (A) presented to primary care with an episode of diarrhoea. Initially treated at home, he subsequently required admission to hospital for management of dehydration. During and following a 24 hour period of hospitalisation, his mother became severely depressed.

**Case presentation:**

A was examined in primary care at aged 11 months, after a referral was received from the well child nurse. A's weight had traversed downwards across four percentile lines and his development had regressed to the age of a 5 – 6 month old.

**Conclusion:**

Incomplete communication between health professionals and lack of follow-up and overall responsibility for A's family resulted in a serious insult to A's physical growth and development. New Zealand needs to adopt a system where one health professional case manages infants and young children in primary care.

## Background

The case of baby A highlights the health risk to infants of having a mother with post natal depression (PND). PND has an estimated prevalence of 13 percent [1]. Meta analyses conducted over the past decade suggest five factors contributing to an increased risk of PND, these include; a history of depression, depression or anxiety during pregnancy, a history of other psychiatric conditions, recent stressful life events and poor social support, particularly the absence of a partner[1]. Baby A's mother had four of these risk factors. Infant mother bonding is crucial in early development, interruptions may be detrimental to the moulding of the hypothalamic pituitary adrenal axis and subsequent brain development[2]. Children of post-nataly depressed mothers are at increased risk of behavioural difficulties, with poor school adjustment and associated social disaffection. Significant links between antenatal anxiety and emotional problems in children, aged four years, were found in the British Avon longitudinal study of parents and children[3]. The South London Child Development study, highlighted that pregnant depressed women were seven times more likely to have children diagnosed with conduct disorder at age 11 than those not depressed [4].

## Case presentation

Baby A was a nine month old baby of mixed New Zealand European and Mâori ethnicity living with his solo mother and 5 year old brother. The family received state financial support. He was a normal delivery at term with a birth weight of 3.5 Kgs (50 percentile). He had no medical history of note. At 9 months old he attended primary care with a history of diarrhoea and reduced feeding for the previous two days. He had a temperature of 37 degrees, weight of 8.5 Kgs (50 percentile) and normal skin turgor. Later that day he was admitted to hospital with worsening diarrhoea and subsequent dehydration, he remained in hospital overnight. The discharge summary to primary care made no reference to the mental health state of A's mother. Three weeks following discharge A was seen by a well child nurse for his 10 month assessment, she observed that A's mother appeared very 'flat'. The nurse faxed a referral requesting an assessment of A and his mother when they visited the surgery later in the week. They did not attend for an appointment. Twelve days following the referral, a child psychologist rang the practice to express her concerns about the mental state of A's mother and the dirty unkempt condition of the house. The psychologist had been visiting A's brother. Following a telephone conversation with A's mother, who stated that she had been unable to care for him very well; she was only feeding him milk and he was spending long periods of time in his cot, an urgent referral was made to the social services department. Baby A was seen and examined 2 days later.

### On examination

A was a 'watchful' baby – there were no smiles or laughs despite protracted efforts to elicit them. He was very 'clingy', resisting all attempts to be laid down on the couch and becoming extremely distressed when his mother attempted to disengage him from her.

**Figure 1:**
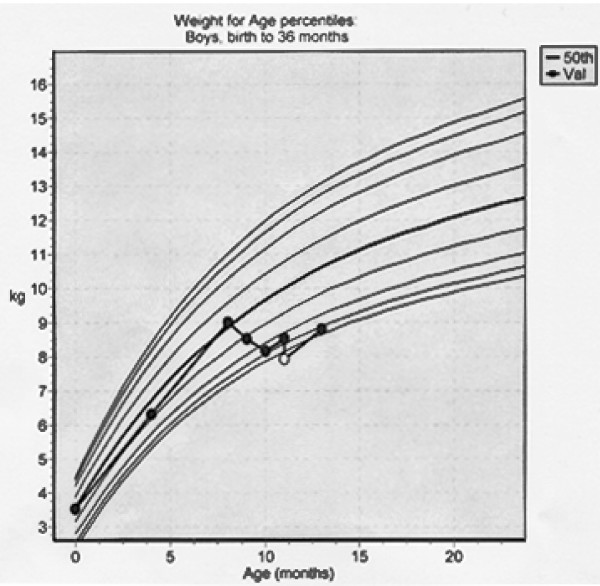
Growth chart of baby A.

Wt: 8.13 Kgs – his weight had traversed downwards across 4 percentile lines (Figure 1). His diet consisted solely of formula milk, 250 ml bottles 5–6 times each day.

Examination of his heart, chest, ears, nose, throat and integument were normal.

He was not sitting, trying to crawl or pulling himself to stand. He could roll. There was no double syllable babble heard during the visit. His mother reported that he cried but didn't babble. He was developmentally at the stage of a 5 – 6 month old.

### Primary diagnosis

Inorganic failure to thrive secondary to neglect.

### Outcome

A's mother was referred to maternal mental health services and is receiving ongoing counselling. The family received parenting support from the social services department. Baby A was seen fortnightly at the surgery for growth and development assessment. At 13 months old he was pulling himself to stand, verbalising double syllable babble, and eating family foods; he was awaiting review by a paediatrician.

## Discussion

Assuming a steady weight gain and no interruptions to A's physical growth, it will take an estimated 6 months for A to 'catch-up' to his original birth weight percentile. There is no way of estimating time for his social and emotional 'catch-up' growth. This critical incident of delayed and incomplete communication between health professionals highlights the need for New Zealand to adopt a system where one health professional case manages infants and young children in primary care.

## Consent

Written informed consent was obtained from the mother of baby A for publication of this case report and accompanying images. A copy of the written consent is available for review by the Editor-in-Chief of this journal.

## Competing interests

The author declares that they have no competing interests.
